# 

**Published:** 1888-05

**Authors:** 


### BOOK NOTICES.

VICK'S AND THE HORTICULTURAL ART JOURNAL, of Rochester, N. Y., are resplendent this month in colored flowers and fruit. We are indebted to both for favors. The gift of seeds from the former, last year, was so prolific of many colored flowers that we passed our hat again this year, and not in vain, which brought wonted smiles to our little petticoated gardeners. Long may they live !

THE CENTURY, like the century plant, has come to full blossom in its art and literary departments. Lincoln, Siberia and other good things are to be found among its leaves for May.

THE YOUTH'S COMPANION.—The very best paper of its kind. Perry Mason & Co., Boston.

We are in receipt of the following publications for which our thanks are due :

SEVENTEENTH ANNUAL REPORT OF THE STATE HOMEOPATHIC ASYLUM for the Insane, Middletown, N. Y. Dr. Selden H. Talcott, Medical Superintendent, author of an excellent essay on the evils of existing laws—properly, *insane* laws.

PRIZE ESSAYS 1, 2, 3 and 4, published by American Public Health Association : No. 1, Healthy Homes ; 2, School Houses and School Life ; 3 and 4, Disinfection and Preventives of Disease, etc., all of great value.

THE PATHOLOGY OF HAY FEVER, by S. S. Bishop, M. D., Chicago.

THE CURABILITY OF EPILEPSY, ETC., by C. H. Hughes, M. D., St. Louis.

THE ASCEPTIC AND ANTESEPTIC MANAGEMENT OF WOUNDS, by David Prince, M. D., Jacksonville, Ill.

INAUGURAL ADDRESS of H. H. Warner before the Rochester Chamber of Commerce, containing valuable statistics of the growth and prosperity of that city, which now numbers 130,000 inhabitants, and other matters of more than a local interest.

### NEW PUBLICATIONS.

A NEW INDEPENDENT MEDICAL AND TEMPERANCE MONTHLY has been launched at Louisville, entitled "The Medical Investigator," under the direction of S. F. Smith, M. D.

AMERICA, 180, 182 Monroe St., Chicago, every Saturday. An excellent paper, full of instructive reading, fit for Sunday or any other day. $3.50 a year.

